# Aberrant Receptor-Mediated Endocytosis of *Schistosoma mansoni* Glycoproteins on Host Lipoproteins

**DOI:** 10.1371/journal.pmed.0030253

**Published:** 2006-07-18

**Authors:** Hein Sprong, Monika Suchanek, Suzanne M van Dijk, Alexandra van Remoortere, Judith Klumperman, Diana Avram, Joke van der Linden, Jeanette H. W Leusen, Jaap J van Hellemond, Christoph Thiele

**Affiliations:** 1 Department of Membrane Enzymology, Bijvoet Center, Utrecht University, Utrecht, Netherlands; 2 Max Planck Institute of Molecular Cell Biology and Genetics, Dresden, Germany; 3 Department of Cell Biology, Utrecht University, Utrecht, Netherlands; 4 Department of Parasitology, Leiden University Medical Centre, Leiden, Netherlands; 5 Department of Biochemistry of Lipids, Utrecht University, Utrecht, Netherlands; 6 Immunotherapy Laboratory, Department of Immunology, University Medical Center Utrecht, Netherlands; 7 Department of Biochemistry and Cell Biology, Institute of Biomembranes, Utrecht University, Utrecht, Netherlands; Free University Medical Center Amsterdam, Netherlands

## Abstract

**Background:**

Bilharzia is one of the major parasitic infections affecting the public health and socioeconomic circumstances in (sub) tropical areas. Its causative agents are schistosomes. Since these worms remain in their host for decades, they have developed mechanisms to evade or resist the immune system. Like several other parasites, their surface membranes are coated with a protective layer of glycoproteins that are anchored by a lipid modification.

**Methods and Findings:**

We studied the release of glycosyl-phosphatidylinositol (GPI)-anchored proteins of S. mansoni and found them in the circulation associated with host lipoprotein particles. Host cells endocytosed schistosomal GPI-anchored proteins via their lipoprotein receptor pathway, resulting in disturbed lysosome morphology. In patients suffering from chronic schistosomiasis, antibodies attacked the parasite GPI-anchored glycoproteins that were associated with the patients' own lipoprotein particles. These immunocomplexes were endocytosed by cells carrying an immunoglobulin-Fc receptor, leading to clearance of lipoproteins by the immune system. As a consequence, neutral lipids accumulated in neutrophils of infected hamsters and in human neutrophils incubated with patient serum, and this accumulation was associated with apoptosis and reduced neutrophil viability. Also, *Trypanosoma brucei,* the parasite that causes sleeping sickness, released its major GPI-anchored glycoprotein VSG221 on lipoprotein particles, demonstrating that this process is generalizable to other pathogens/parasites.

**Conclusions:**

Transfer of parasite antigens to host cells via host lipoproteins disrupts lipid homeostasis in immune cells, promotes neutrophil apoptosis, may result in aberrant antigen presentation in host cells, and thus cause an inefficient immune response against the pathogen.

## Abstract 2

The finding that GPI-anchored schistosome proteins are transferred from the parasite surface to human lipoproteins may explain how the parasites interfere with an effective immune response.

## Editors' Summary

**Background.:**

More than 200 million people live in a close but uneasy alliance with schistosomes, a type of parasitic worm. Like many parasites, schistosomes have a complicated life cycle. They start life by reproducing in fresh-water snails. The snails release free-swimming, infectious parasites, which burrow into the skin of people who swim in the water. The parasites then migrate to the veins draining the gut and mature into 10–20 mm-long adult worms. The worms mate and lay eggs, some of which pass into the feces and so back into water where they hatch and infect fresh snails. Schistosomiasis does not kill many people but it does cause serious health problems. Most of these are caused by the human immune system responding to eggs that get trapped in the veins of the liver, spleen, and gut. Immune cells recognize proteins on the eggs as foreign and organize a hard shell of immune cells and tough fibres around the egg. Eventually, these fibres block the blood vessels in the liver, spleen, and gut, causing locally raised blood pressure, organ damage, and potentially fatal bleeding.

**Why Was This Study Done?:**

Although the immune system mounts a vigorous attack against schistosome eggs, the parasites themselves somehow evade the immune response—adult worms pull off this feat of “invisibility” for years. The researchers who did this study wanted to find out whether the release of glycoproteins (proteins decorated with sugars) from the surface of the schistosome worms is involved in this immune evasion in some way. These glycoproteins (which are anchored to the parasite's surface by a structure called a GPI-anchor; GPI stands for glycosyl-phosphatidylinositol, a sort of fat or lipid) are the major antigens of schistosomes—the molecules that the immune system normally recognizes on foreign intruders.

**What Did the Researchers Do and Find?:**

The researchers first showed that GPI-anchored schistosomal glycoproteins are released into the circulation of patients and there become attached to human lip oproteinparticles (water-soluble carrier molecules that take fats around the body). Then, using cells grown in the laboratory, the researchers discovered that lipoprotein particles loaded with parasite glycoproteins could enter mammalian cells through an interaction with a protein called the low-density lipoprotein receptor, which normally helps cells absorb the lipids needed to make membranes. Once in the cell, the parasite glycoproteins travelled to cellular regions called lysosomes, which they seemed to disrupt. In addition, the researchers found that the parasite glycoproteins could enter mammalian cells by a second route: This involved the glycoproteins being taken up by neutrophils (a type of immune cells). Many of these neutrophils then died, possibly because of the large amount of lipid they accumulated.

**What Does This Mean?:**

These results provide some tantalising clues to how schistosomes might evade the immune response. First, just binding to lipoprotein particles might change how they are seen by the immune system (possible they are not as clearly recognized as foreign substances) and weaken the immune response against them. On the other hand, the damage done to neutrophils by lipid accumulation might also contribute to how schistosomes hide in the human hosts. Neutrophils are an important type of immune cell, and their destruction could compromise the immune system's response to schistosomes. Furthermore, although the researchers do not investigate this possibility, other cells of the immune system that have might also take up these lipids and be damaged. Finally, even if immune cells are not killed outright by lipid accumulation, disruption of their lysosomes might also affect how well the immune system recognizes schistosomes as foreign. The full details of the complex interplay between schistosomes and their hosts remain a mystery, but these results provide intriguing new avenues to explore that might eventually suggest new treatments for schistosomiasis.

**Additional Information:**

Please access these websites via the online version of this summary at http://dx.doi.org/10.1371/journal.pmed.0030253.

• World Health Organization information on schistosomiasis

• US Centers for Disease Control and Prevention information for the public and for professionals on schistosomiasis

• MedlinePlus encyclopedia entry on schistosomiasis

• Wikipedia page on schistosomiasis (note: Wikipedia is a free online encyclopedia that anyone can edit)

## Introduction

Schistosomiasis, also known as bilharzia, is a chronic disease caused by parasitic *Schistosoma* flatworm species [1] that affects more than 200 million people worldwide. The schistosome is a prime example of a complex multicellular organism that can flourish in human hosts for decades despite the development of a pronounced immune response. The molecular basis for the prolonged survival of adult schistosomes is an outstanding issue [2].

The major surface antigens of adult schistosomes are tightly associated with the extracellular leaflet of the outer membranes by a glycosyl-phosphatidylinositol (GPI) anchor [3,4]. GPI-anchored proteins are abundant in parasites and have an important role in cell viability and defence against the host immune system [5,6]. They can be shed into the medium by the action of proteases or phospholipases [7] or released on membranous particles [8−11]. A third and largely unexplored mechanism is their release on lipoprotein particles. Lipid-modified proteins on the exoplasmic face of the plasma membrane might fit well into the phospholipid monolayer of such a particle, as demonstrated by the presence of GPI-anchored CD59 in HDL particles [12]. These lipoprotein particles might further transfer GPI-anchored proteins onto other cells, resulting in a net intercellular transfer. Interestingly, when transfected into cultured cells, GPI-anchored proteins can be transferred between cells [13−16]; this process was also observed in mice transgenic for human CD59 [17]. Trypanosome surface GPI protein has been observed on host erythrocytes [18], and retrograde transfer of host GPI protein onto the parasite was found in schistosomes [19]. It is not known how this transfer works mechanistically nor whether lipoprotein particles are involved.

We recently found that the lipid-linked morphogens Hedgehog and Wingless associate with *Drosophila* lipoprotein particles, and that their intercellular transport depends on the presence of lipoproteins [20]. Similarly, parasites such as schistosomes might utilize host lipoprotein particles as carriers for their GPI-linked surface antigens. Here, we demonstrate that parasite GPI-anchored glycoproteins are released onto host lipoprotein particles that are taken up by host cells via the low-density lipoprotein (LDL) receptor and the Fc receptors. This intercellular transfer of parasite GPI-anchored proteins to its host has important pathophysiological implications.

## Methods

### Materials

Chemicals, unless indicated otherwise, were from Sigma (St. Louis, Missouri, United States) and used in the highest purity available. Silica TLC plates were from Merck (Darmstadt, Germany), organic solvents were from Riedel de Haën (Darmstadt, Germany), and cell culture media and reagents were from Invitrogen (Breda, The Netherlands). Cell culture plasticware was from Costar (Cambridge, Massachusetts, United States). Phosphatidylinositol-specific phospholipase C (PI-PLC) from Bacillus cereus was from Molecular Probes (Eugene, Oregon, United States). Tran[^35^S]label (>36 TBq/mmol) was from ICN (Costa Mesa, California, United States). [U-^14^C]palmitic acid (18 TBq/mol) was from Amersham (Buckinghamshire, UK). Jürgen Gent generously provided us with a plasmid containing the human LDL receptor.

### Antibodies

The synthetic peptide CIKAHDYKLLTKILAARQLQDLFDNDKN, which corresponds to amino acids 29–55 from the GPI-anchored surface protein SM200 of S. mansoni [21], was conjugated with its amino-terminal cysteine to keyhole limpet haemocyanin, and used for immunization of rabbits (Eurogentec, Seraing, Belgium). Antibodies to SM200 were affinity purified using the same peptide coupled to Affigel-15 columns (BioRad, Hercules, California, United States). The mouse monoclonal antibody (mab) to SM200, 307D5 [4], was kindly provided by Karl Hoffmann (University of Cambridge, UK). The mouse mab 120–1B10 to circulating anodic antigen (CAA) from S. mansoni—which specifically recognizes schistosomal glycoproteins (sGPs) carrying O-linked GlcAβ1–3GalNacβ1–6(GlcAβ1–3)GalNAcβ1 repeating units—was described previously [22,23]. The mouse mab 114–5B1, which recognizes the schistosome-specific oligosaccharide structure GalNacβ1–4[Fucα1–2Fucα1–3]GlcNacβ-R (or LDN-DF), was described previously [24,25].

Polyclonal rabbit antisera recognizing the human LDL receptor [26] and calreticulin [27] were a generous gift of Jürgen Gent and Ineke Braakman (Utrecht University). Blocking antibody against human FcγRIIa was mab IV.3 from Medarex (Annandale, New Jersey, United States). Rabbit anti-human ApoA1 and ApoB were from Calbiochem (La Jolla, California, United States), rabbit anti-mouse ApoA1 was from Biodesign (Saco, Maine, United States). Rat mab 1D4B to mouse LAMP-1 was from Santa Cruz Biotechnology (Santa Cruz, California, United States). Biotinylation of affinity-purified antibodies with biotin-X succinimidyl ester (Molecular Probes) was performed according to the vendor's instructions. Fluorescently labelled secondary goat antibodies were obtained from Jackson ImmunoResearch Laboratories (West Grove, Pennsylvania, United States). Horseradish peroxidase-conjugated secondary goat antibodies were from DAKO (Glostrup, Denmark).

Antibodies used in immuno-electron microscopy were anti-120–1B10-biotin conjugate, rabbit anti-SM200-biotin, rabbit anti-apolipoprotein B, goat anti-biotin conjugated to 10 nm gold (Aurion, The Netherlands) and 10 nm protein A-gold (Cell Microscopy Center, Utrecht, The Netherlands)

### Sera

Sera of S. mansoni-infected individuals were obtained from the WHO/TDR Reference Serum Bank for African Schistosomiasis. Control sera were from donors with no known history of schistosomiasis.

Heat-inactivated foetal calf serum (FCS) was delipidated (DL-FCS) by solvent extraction. Serum was mixed with an equal volume of a 2:1 mixture of diisopropyl ether:*n*-butanol, stirred at room temperature for 30 min, and phases were separated by centrifugation at 5,000 *g* for 30 min. The aqueous phase was re-extracted as before with an equal volume of diisopropyl ether, dialyzed against PBS, and filter-sterilized. This treatment resulted in the total loss of intact lipoprotein particles, as judged by the loss of apolipoprotein A and B from the top fraction of a KBr gradient centrifugation analysed by SDS-PAGE and Coomassie brilliant blue staining. The cholesterol content was reduced to less than 5% of untreated serum, as determined by a colorimetric assay kit (Boehringer-Mannheim, Indianapolis, Indiana, United States).

### Cell Culture, Transfection, and Uptake Experiments

Peripheral blood from normal human volunteers was collected in heparin tubes, and polymorphonuclear neutrophils (PMNs) were isolated by density gradient centrifugation using Ficoll-Hypaque [28]. The neutrophils were divided into 4-ml samples with 10^6^ cells/ml in RPMI 1640 medium containing 25 mM Hepes (pH 7.2), 2 mM nonessential amino acids, 1 mM sodium pyruvate, 2 U/ml interferon γ, and 20% heat-inactivated human serum. After cells were incubated for 8 h with 20% patient or control sera at 37 °C with 5% CO_2_, they were washed extensively with ice-cold PBS, lysed in reducing Laemmli sample buffer, and subjected to Western blotting.

Mutant Chinese hamster ovary cells lacking the mature LDL receptor (LDLA cells) [29] were kindly provided by Ineke Braakman (Utrecht University, the Netherlands) and grown in Ham's F12 medium with 5% FCS, 2 mM glutamine at 37 °C with 5% CO_2_. LDLA cells were transfected with the human LDL receptor in pCDNA3 [26] or the empty vector using Lipofectamine 2000. Transfectants were cultured in normal culture medium containing 1 mg/ml geneticin. Stable cell lines were obtained by subcloning individual colonies. Positive clones were selected by immunofluorescence microscopy and tested for expression of hLDL-R by Western blotting. LDLA cells were transfected with the human FcγRIIa expression vector FcγRIIa-pRC-CMV [30] or the empty vector using Lipofectamine 2000. Expression of FcγRIIa was induced by 5 mM sodium butyrate 14–16 h prior to experiment. For uptake experiments, confluent cells on 3-cm dishes, or subconfluent cells on glass coverslips, were incubated with Ham's F12 medium containing 2 mM glutamine and 20% human serum for 6 h at 37 °C with 5% CO_2_. Cell were washed extensively with ice-cold PBS and subjected to either Western blotting or immunofluorescence microscopy as described [31], except that the cells were examined with a Nikon D-eclipse C1 confocal microscope using separate filters for each fluorochrome viewed (FITC: L_ex_ = 488 nm and L_em_ = 515 LP; Texas red: L_ex_ = 568 nm and L_em_ = 585 LP). Single-labelled cells with each primary/secondary antibody combination were examined and confirmed that no bleed-through occurred for the given conditions.

### Metabolic Labelling of Adult S. mansoni Worms

S. mansoni adult worms were collected by perfusion of the hepatic portal system of golden hamsters at 7 wk after infection with ~ 500 cercariae per hamster [32]. About 100 adult worms pairs were incubated with 5 ml RPMI 1640 medium containing 100 mM Hepes (pH 7.2), 2 mM sodium pyruvate, 100 U/ml penicillin, and 100 μg/ml streptomycin. For radiolabelling of worm proteins and lipids, 18 MBq/ml Tran[^35^S]-label and 18 kBq/ml [U-^14^C]palmitic acid were added to the medium, respectively. After worms were incubated for 24 h at 37 °C with 5% CO_2_, the medium was subjected to isopycnic density centrifugation. The worms were washed with ice-cold PBS, and the worm proteins and lipids were analysed as described below.

### Neutrophil Apoptosis and FACS Analysis

PMNs were isolated from healthy volunteers as described and cultured in RPMI 1640 medium containing 20% control or patient serum, or 10% serum preincubated with 10 mg of rabbit anti-ApoB antibodies or rabbit IgG (control) for 30 min at room temperature, and immunocomplexes were allowed to form. After 20 h of culture, cells were counted and washed in binding buffer consisting of 10 mM Hepes, 150 mM NaCl, 5 mM KCl, 1.8 mM CaCl_2_, and 1 mM MgCl_2_. Annexin V FITC (BD Biosciences Pharmingen) was added to the cells and incubated for 15 min at room temperature, and cells were measured on a FACSCalibur (Becton Dickinson, San Jose, California, United States) to determine the percentage of dead cells. Survival was calculated as the percentage of living cells relative to the input number of PMNs.

### Isopycnic Density Centrifugation and PI-PLC Treatment

Serum or medium was spun for 3 h at 39,000 rpm at 4 °C in a SW41 rotor (Beckman). Pellet and supernatant were designated as P120 and S120, respectively. In some cases, the S120 was split in two equal fractions and either treated with 4 U/ml PI-PLC or mock-treated for 6 h at 37 °C. Lipoprotein particles were separated from soluble proteins by isopycnic density centrifugation. In short, KBr was added to S120 to a final concentration of 0.33 g/ml, and the sample was spun for 2 d at 39,000 rpm at 4 °C in a SW41 rotor. Six or twelve fractions were taken from the top. The top and bottom fractions had a density of 1.22 and 1.40 g/cm^3^, respectively. The proteins were precipitated with chloroform/methanol, and the lipids in the chloroform phase were analysed separately. Lipids were separated by thin-layer chromatography using silica gel 60 plates and chloroform/methanol/25% NH_4_OH (65:25:4 v/v) as running solvent. Radiolabelled spots were detected by phosphor-imaging on a Storm phosphor-imager. Spots were identified by comparison to standards and quantified using the Imagequant Software (Molecular Dynamics, Sunnyvale, California). Radiolabelled proteins were analysed by SDS-PAGE followed by autoradiography or by Western blotting.

### Immunoprecipitation

S120 was precleared during a 2-h incubation with 0.25 volumes of streptavidin-agarose or empty Sepharose beads. A fraction of each supernatant was used to determine relative amounts of ApoA1 and ApoB by Western blotting. The remainder was incubated with biotinylated anti-rabbit SM200 or a mixture of biotinylated 120–1B10 and 114–5B1 antibodies preadsorbed to streptavidin-agarose. For immunoprecipitations of human antibodies from serum, the samples were incubated with protein A-Sepharose beads without addition of a primary antibody. Immunoprecipitates were washed at least ten times with five volumes of PBS containing 0.5% bovine serum albumin and once with five volumes of PBS. Proteins were eluted from beads using either a detergent-containing buffer (150 mM NaCl, 2 mM EDTA, 100 mM Tris-Cl [pH 8.3], 0.5% Nonidet P 40, 0.5% sodium deoxycholate, and 0.1% SDS) or Laemmli sample buffer containing 5% β-mercaptoethanol.

### Immuno-Electron Microscopy

Lipoprotein particles were separated from the blood of either the positive or the control patient by a KBr gradient. Of this fraction 3-μl droplets were placed on carbon-coated copper grids with a formvar film and incubated for 30 min. The particles attach to the formvar film. After washing with PBS, the particles were labelled as previously described [33] with biotinylated primary antibodies followed by goat anti-biotin directly coupled to gold. To detect human antibodies on host lipoproteains, purified particles were labelled with protein A-gold only.

## Results

### Schistosomes Release Proteins and Lipids on Lipoprotein Particles

**Figure 1:**
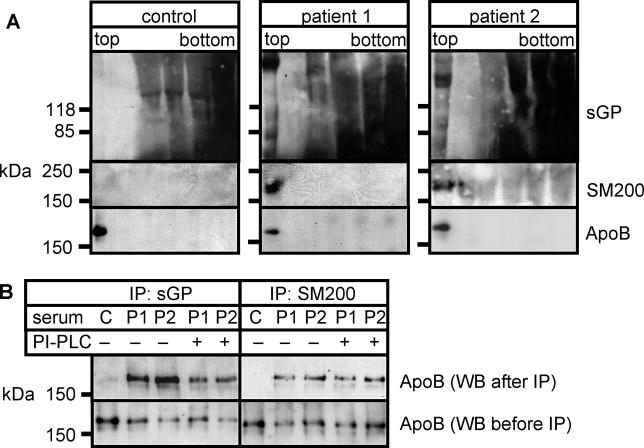
Lipoprotein Particles of Infected Humans Contain Schistosomal Surface Proteins (A) Lipoprotein particles of control and patient sera were separated from soluble proteins by KBr density gradient centrifugation. Proteins from gradient fractions were analysed by SDS-PAGE and Western blotting against schistosome-specific glycoproteins (sGP), schistosomal GPI-anchored protein SM200, and human apolipoprotein B (ApoB). Note the presence of sGP and SM200 in the top fractions that contain the lipoprotein particles. Strong background staining is due to high protein concentrations in serum; data are representative of six different patient sera. (B) Control (C) and patient (P1 and P2) sera were mock-treated (−) or treated with PI-PLC (+) and subjected to immunoprecipitation with biotinylated antibodies against either sGP (left) or SM200 (right). Immunocomplexes were isolated with streptavidin-agarose beads and analysed by SDS-PAGE and Western blotting for ApoB. Streptavidin beads alone did not pull down ApoB in patient sera (unpublished data). Antibodies were raised against a peptide sequence or a common glycoconjugate and recognize the protein irrespective of the presence of the diacylglycerol moiety.

Adult schistosomes release proteins and lipids from their surface into the circulation [34]. To investigate whether they are present on lipoprotein particles, sera from patients chronically infected with S. mansoni were cleared of parasite remnants and membrane fragments by high-speed centrifugation and analysed by isopycnic density centrifugation (Figure 1A). The top fraction contained all lipoprotein particles, whereas soluble proteins were found in fractions of relatively higher density. Remarkably, several GPI-anchored sGPs as well as the GPI-anchored surface protein SM200 selectively accumulated in the lipoprotein fraction of patient but not control sera (Figure 1A, top lanes).

Next, we tested whether ApoB, the major protein constituent of human LDL particles, could be coimmunoprecipitated with antisera against sGP or SM200. To avoid effects resulting from the presence of anti-*Schistosoma* antibodies in patient serum (see below), we used biotinylated antibodies and streptavidin-agarose for pull-downs throughout. ApoB was coimmunoprecipitated from patient sera, but not from control serum, by antibodies against both schistosomal antigens (Figure 1B), indicating a physical association of schistosomal GPI-anchored proteins with human lipoprotein particles. The association of sGP was sensitive to treatment with PI-PLC (Figure 1B, left), suggesting that they bound to lipoprotein particles via the GPI anchors. Immunoprecipitation of the GPI-anchored protein SM200 after PI-PLC treatment did not result in a significant reduction of recovered ApoB (Figure 1B, right), consistent with a previous report that the GPI anchor of SM200 is resistant to PI-PLC [35].

**Figure 2:**
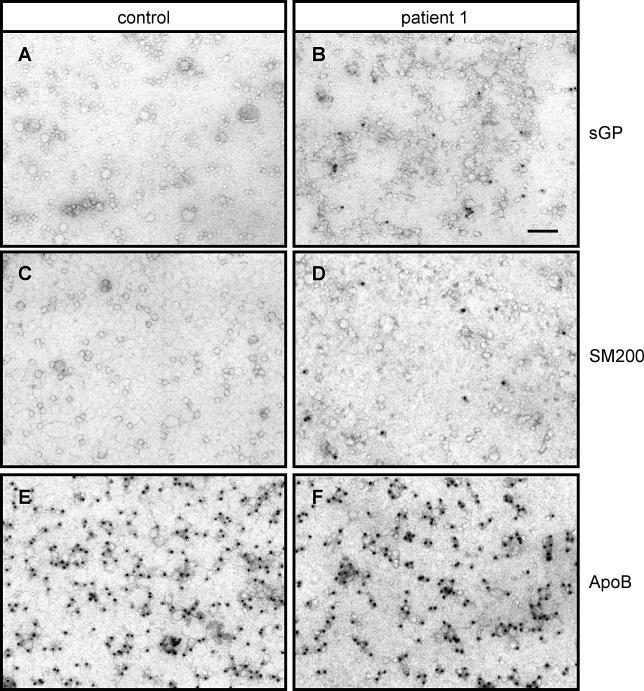
Schistosomal GPI-Linked Surface Proteins Directly Bind to Lipoprotein Particles Lipoprotein particles isolated from control (A, C, and E) and patient (B, D, and F) sera were stained by immunogold labelling for schistosome-specific glycoconjugates using biotinylated antibody 120–1B10 (A and B), anti-SM200 (C and D), and anti-ApoB (E and F) and goat anti-biotin conjugated to 10 nm gold and visualized by electron microscopy. Bar, 100 nm. Data are representative of three infected individuals.

The localization of schistosomal proteins on lipoprotein particles was further assessed by electron microscopy (Figure 2). Lipoprotein particles of 10–60 nm diameter were detected in both control and patients sera by immunostaining against ApoB (Figure 2E and 2F). No membranous fragments or parasite remnants were detected in any of the samples. Purified lipoprotein particles from patient serum contained both sGP and SM200 (Figure 2B and 2D), which were lacking in the control sample (Figure 2A and 2C). Note the close proximity of gold particles and lipoprotein particles; no gold dot is found that is not associated with a particle, ruling out background binding to nonparticulate material or to a possible soluble, free form of the antigen. We performed statistical analysis on the data in Figure 2A and 2B. In three independent fields of different patients or control individuals we counted total lipoprotein particles (altogether 9,700 in control and 5,900 in patient fields) and gold particles (two controls, 48 patients) to give values of 0.5 ± 0.5 gold/1,000 lipoprotein particles for control and 8.2 ± 2 gold/1,000 lipoprotein particles for patients (*p* < 0.001).

### Transfer of Schistosomal Proteins onto Lipoprotein Particles In Vitro Depends on Intact Host Lipoprotein Particles

**Figure 3:**
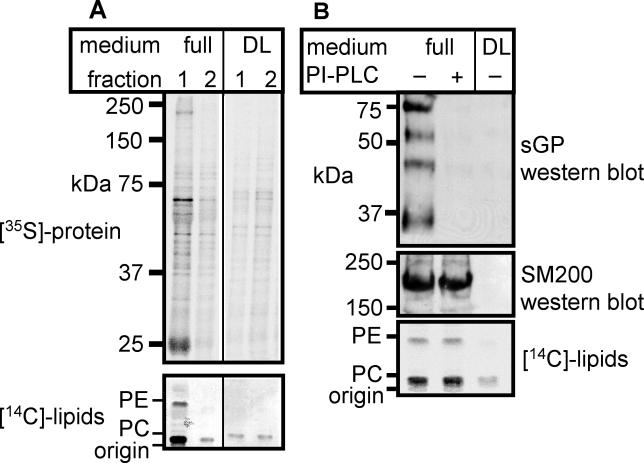
Schistosomes Transfer Glycoproteins and Lipids to Lipoprotein Particles In Vitro Adult S. mansoni worms were incubated overnight in medium with [^35^S]-amino acids and [^14^C]-palmitic acid in the presence of normal (full) or delipidated (DL) FCS, and media were precleared by high-speed centrifugation. (A) Media were subjected to KBr density gradient centrifugation, and fractions were analysed for schistosomal proteins by SDS-PAGE followed by autoradiography (top) and for schistosomal lipids by TLC followed by autoradiography (bottom). The top and second top fractions of the gradients are shown. Note the presence of prominent protein bands at 25, 70, and 250 kDa in the top fraction of full but not of delipidated medium. (B) Top fractions from KBr density gradient centrifugations of mock-treated (− PI-PLC) or PI-PLC treated (+ PI-PLC) normal media (full) or delipidated media (DL) were analysed for schistosome-specific glycoproteins (top) and for SM200 (middle) by Western blotting and for schistosomal lipids by thin-layer chromatography followed by autoradiography (bottom).

To reconstitute the transfer of GPI-anchored proteins onto lipoprotein particles outside the host organism, we kept adult worms in culture medium supplemented with [^35^S]Cys/Met and [^14^C]palmitate and analysed the medium for radiolabelled parasite proteins and lipids. After worms and membrane fragments were removed by high-speed centrifugation, about 2% of the radioactive proteins were found in the high speed supernatant S120 (unpublished data). The lipoprotein fraction was separated from the rest of the S120 by isopycnic density centrifugation on a KBr gradient. Figure 3A shows the two top gradient fractions of the experiment done in normal or in delipidated medium; lipoproteins accumulate in the very top fraction 1. At least six distinct parasite proteins were found in this lipoprotein particle fraction (Figure 3A, full, fraction 1), suggesting association with lipoprotein particles. Incubation of schistosomes in solvent-extracted delipidated medium, free of lipoprotein particles, resulted in a loss of parasite proteins from the top fraction of the gradient (Figure 3A, DL, fraction 1). Schistosomal lipids, synthesized from the added [^14^C]palmitate, were also found in the top fraction of the full, but not the delipidated medium (Figure 3A, [^14^C]-lipids). This indicates that S. mansoni transfers not only protein but also lipids onto host lipoprotein particles. Mass spectrometrical analysis confirmed the presence of schistosome-specific C20:1 fatty acid on phosphatidylcholine in the lipoprotein fraction of patient, but not of control serum (unpublished data).

Both SM200 and sGP were found in the lipoprotein fraction of the same culture medium (Figure 3B), indicating that at least some of the radioactive schistosomal proteins in the lipoprotein fraction are GPI-anchored. This association was sensitive to PI-PLC treatment prior to density centrifugation, which caused sGP, but not SM200, to disappear from the top fraction (Figure 3B, full +), consistent with the observations made with patient sera (Figure 1). We conclude that the transfer of GPI-linked schistosomal surface proteins to lipoprotein particles can take place in vitro, requires the presence of host lipoprotein particles in the serum, and is independent of lipoprotein turnover by the host cells.

### LDL Receptor-Dependent Uptake of Schistosomal Glycoproteins

**Figure 4:**
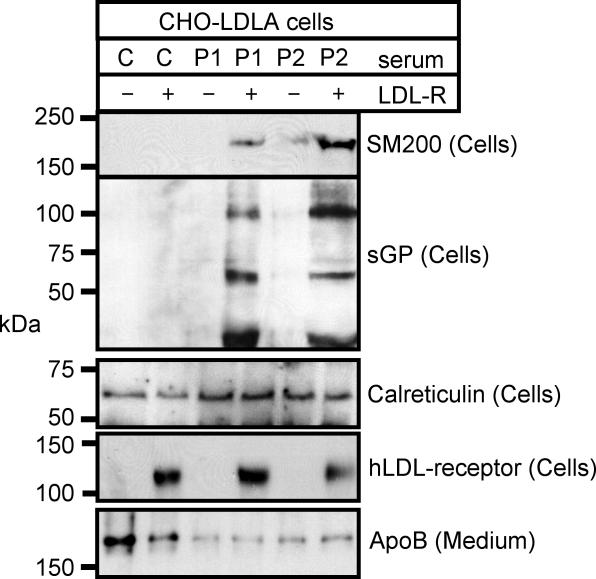
The LDL Receptor Pathway Can Mediate Endocytosis of Schistosomal Surface Proteins CHO-LDLA cells lacking (−) or retransfected with (+) LDL receptor were incubated with control (C) or patient (P1 and P2) sera. Cell lysates were subjected to SDS-PAGE and Western blotting for endocytosed SM200 or schistosome-specific glycoproteins. As loading controls, the medium was probed for ApoB, and the cells for calreticulin and for the presence of transfected LDL-receptor.

**Figure 5:**
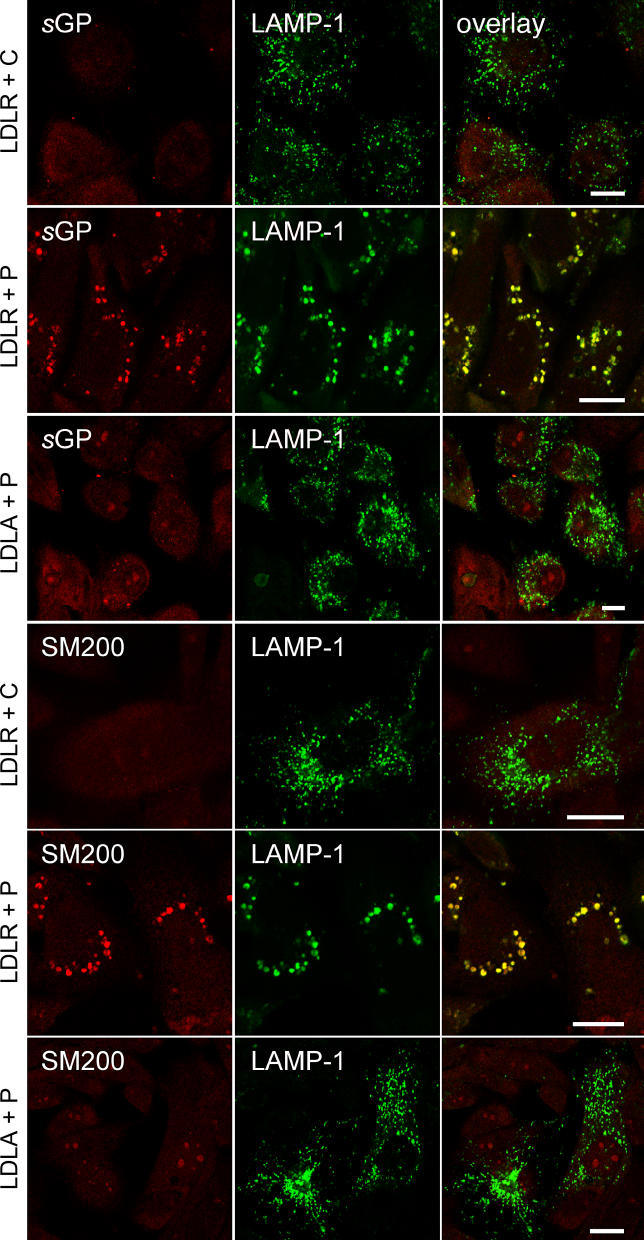
Endocytosed Schistosomal Surface Proteins Accumulate in Enlarged Lysosomes CHO-LDLA cells lacking (LDLA) or re-transfected with LDL receptor (LDLR) were incubated with control (C) or patient (P) sera, fixed, and processed for immunofluorescence microscopy. Localization of schistosomal glycoproteins (sGP, red) or SM200 (SM200, red) and of the lysosomal marker protein LAMP-1 (green) was imaged by confocal laser fluorescence microscopy. Note the enlarged lysosomes in cells (LDLR + P) that take up schistosomal proteins on lipoprotein particles. Bars, 10 μm.

To distribute lipids to peripheral tissues, LDL particles are constantly endocytosed by recipient cells. Thus, schistosomal antigens on lipoprotein particles should spread not only in the circulation, but also into host cells in the periphery of the body. To address this possibility, CHO-LDLA cells, which lack a functional LDL receptor [29], were stably retransfected with the human LDL receptor or were mock-transfected and incubated with medium containing control (C in Figure 4) or patient (P1 and P2 in Figure 4) serum. As shown by immunoblotting of cell lysates, only cells with the LDL receptor (+ in Figure 4), but not mock-transfected controls (− in Figure 4) were able to take up SM200 and other schistosomal glycoproteins from patient serum. To follow the intracellular localization of the internalized material, confocal immunofluorescence microscopy was performed (Figure 5). Cells with (LDLR in Figure 5) or without (LDLA in Figure 5) the LDL receptor were incubated in patient serum, then stained for either sGP or SM200 (red) and counterstained for the lysosomal marker protein LAMP-1 (green). As a negative control, cells with LDL receptor were also incubated in control serum and stained for the same markers. Both sGP and SM200 became internalized into structures positive for the lysosomal marker protein LAMP-1 (LDLR + P in Figure 5), as demonstrated by a virtually complete colocalization of the two markers (overlay, yellow). These lysosomes were characteristically increased in size (diameter mean ± standard deviation: control, 0.4 ± 0.2 μm; patient serum, 1.0 ± 0.3 μm; *n* = 38 each; *p* < 0.001) and decreased in number compared to cells that either expressed no receptor (Figure 5, LDLA + P) or expressed receptor but received control serum (Figure 5, LDLR + C). A small amount of schistosomal antigens was also internalized in LDLA cells (Figure 5, LDLA + P), but did not colocalize with the lysosomal marker.

### Antibody-Antigen Complexes on Lipoprotein Particles in Patient Serum

**Figure 6:**
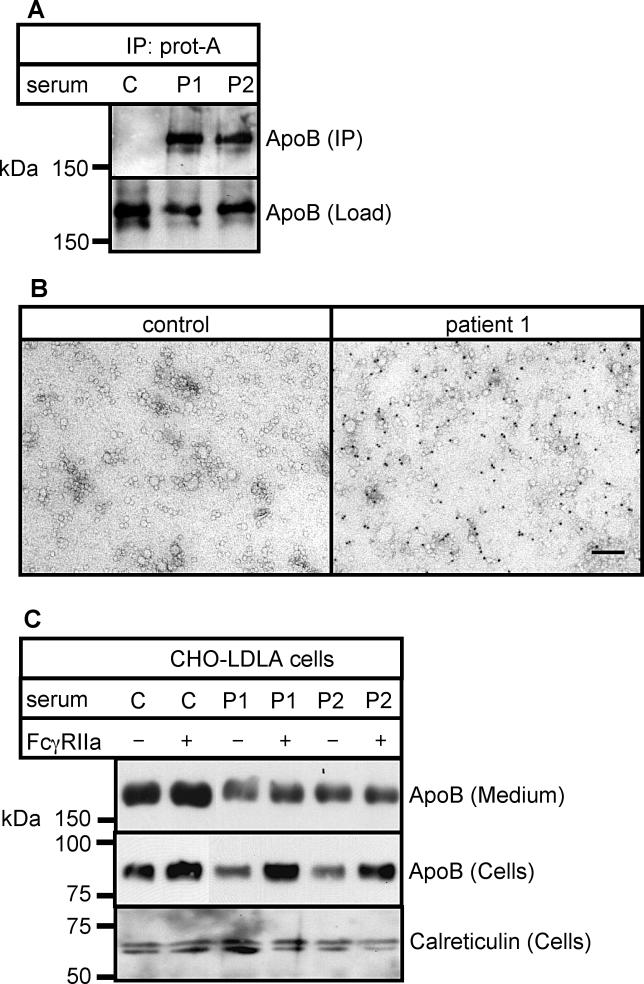
Lipoproteins from Infected Humans Are Decorated with Antibodies and Can Be Endocytosed Via the FcγRIIa Receptor (A) Control (C) and patient (P1 and P2) sera were incubated with protein A-Sepharose beads. Bound material was eluted and subjected to SDS-PAGE and Western blotting for ApoB. (B) Lipoprotein particles were isolated from control and patient sera, stained for endogenous antibodies present on lipoprotein particles using protein A-gold, and subjected to electron microscopy. (C) CHO-LDLA cells lacking (−) or transfected with (+) FCγRIIa receptor were incubated with control (C) or patient (P1 and P2) sera. Cell lysates were subjected to SDS-PAGE and Western blotting for endocytosed ApoB (ApoB [Cells]). A proteolytic fragment [56] of ApoB was detected at ~80 kDa. As load controls, the medium was probed for ApoB (ApoB [Medium]), and the cells for calreticulin.

The presence of schistosomal antigens on host lipoprotein particles enables host antibodies to bind indirectly to lipoproteins via the attached antigens. In patient serum, but not in control serum, ApoB was precipitated by IgG-binding protein A beads without the addition of antibodies against schistosomal antigens, demonstrating binding of host antibodies to lipoprotein particles (Figure 6A). Ultrastructural analysis (Figure 6B) also showed human IgG on lipoprotein particles from patient serum.

This result suggests that cells expressing low-affinity IgG receptors such as FcγRIIa could specifically bind and endocytose liporotein particles from infected patients. To study this possibility, we transfected CHO-LDLA cells with FcγRIIa and analysed the amount of ApoB that was endocytosed from control and patient serum (Figure 6C). Presence of the receptor caused an increase in endocytosed lipoprotein particles from patient but not from control serum.

### Neutrophils Accumulate Lipids from Patient Serum by IgG Receptor-Mediated Lipoprotein Uptake

**Figure 7:**
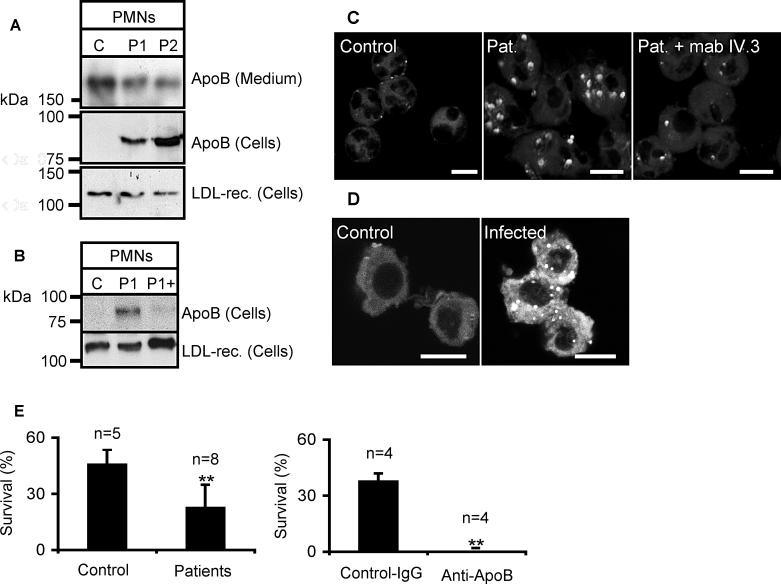
Lipoproteins of Patient Serum Are Endocytosed by Neutrophils and Lead to Intracellular Accumulation of Neutral Lipids and Enhanced Neutrophil Apoptosis (A, B, and C) Freshly isolated human PMNs were incubated for 16 h with control (C) and patient (P, P1, P2, and Pat) sera. For (A and B), cells were washed extensively and cell lysates probed for ApoB and LDL-receptor by SDS-PAGE and Western blotting. A proteolytic fragment [56] of ApoB was detected at ~80 kDa. LDL receptor served as a load control. For (B and C), the FcγRIIa blocking mab IV.3 was added during the incubation (P1+ [B]; Pat. + mab IV.3 [C]). For (C), cells were fixed, stained with Nile Red and subjected to confocal fluorescence microscopy. (D) PMNs of control or schistosome-infected hamster were stained with Nile Red and subjected to confocal fluorescence microscopy. Data are representative of four infected and control animals. (E) PMNs were incubated for 20 h with 20% (left) or 10% (right) control or patient serum as indicated. For the right bar graph, 10 μg/ml normal rabbit antibody (Control) or anti-ApoB antibody were preincubated with control serum, and subsequently added to PMNs. Cells were counted before and after incubation with serum, stained with Annexin V-FITC (which indicates apoptosis), and analysed by FACS. Results are expressed as percentage of Annexin V-negative cells, relative to the number of cells before incubation with serum. Statistical analysis was done by Student's t-test, ** *p* < 0.01.

Human PMNs physiologically express the FcγIIRa receptor and might endocytose antibody-lipoprotein complexes from schistosomiasis patient serum. We found that freshly isolated human neutrophils took up lipoprotein particles from patient serum (P1 and P2 in Figure 7A), but not from control serum (Figure 7A and 7C). This uptake actually depended on the FcγIIRa receptor, since it could be blocked by addition of a blocking antibody, mab IV.3 [36], against this receptor (Figure 7B). This result suggests that clearance by the immune system is one of the reasons for the low levels of lipoprotein particles observed in schistosomiasis patient circulation [37].

Uptake of lipoprotein particles by neutrophils delivers not only apolipoproteins but also the associated lipids, which could affect the cells' lipid balance. To address this question, we stained neutrophils that had been incubated in control or patient serum with a dye, Nile red, that specifically detects accumulations of neutral lipid. Increased accumulations of neutral lipid in multiple round structures, likely lipid droplets, were found after incubation in patient serum (Pat, Figure 7C), again inhibited by the presence of mab IV.3 during the incubation (Pat + mab IV.3, Figure 7C). To test if this neutral lipid accumulation also occurs in the acute disease state, we isolated PMNs from control or S. mansoni-infected hamsters and examined these cells by Nile red staining. We found an accumulation of lipid in cells of infected but not of control animals (Figure 7D).

If this uptake of antibody-antigen-lipoprotein complex were harmful to the neutrophils, it might contribute to the parasites' evasion of host immune response. We cultured freshly isolated PMNs with control or patient serum, which led to apoptosis of a major fraction of the cells under both conditions, with a significantly reduced survival in patient serum (left bar graph, Figure 7E). To test whether reduced survival could be caused by antibody-lipoprotein complexes, we repeated the experiment in normal serum that was preincubated with control antibodies or anti-ApoB antibodies, to allow anti-ApoB antibodies to bind directly to lipoprotein particles. This treatment resulted in nearly complete apoptosis (right bar graph, Figure 7E), suggesting that antibody-lipoprotein complexes indeed destroy the most abundant phagocytes in the blood and thereby inhibit the immune response.

### Presence of GPI-Linked Surface Antigens on Lipoproteins is a Widespread Phenomenon

**Figure 8:**
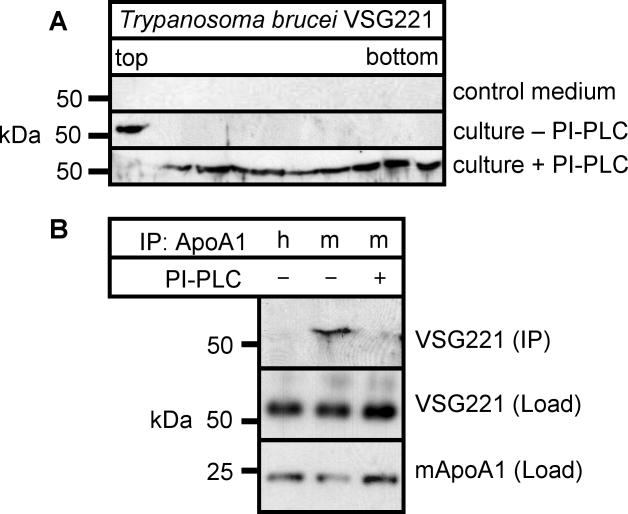
Variant Surface Glycoprotein VSG221 of T. brucei Associates with Lipoprotein Particles (A) T. brucei was cultured in vitro in the presence of mouse serum. Control medium (control) and medium from T. brucei in vitro cultures that was treated with PI-PLC (VSG221 + PI-PLC) or left untreated (VSG221) was subjected to KBr density gradient centrifugation. Gradient fractions were analysed by SDS-PAGE and Western blotting for the presence of VSG221. (B) Medium from T. brucei in vitro cultures was treated with PI-PLC (+) or left untreated (−) and subjected to immunoprecipitation using anti-human (h) or anti-mouse (m) antibodies against ApoA1. Immunoprecipitates were subjected to SDS-PAGE and probed for VSG221. The lower blots shows loading controls for VSG221 and mouse ApoA1 from nonimmunoprecipitated medium.

The potential to release GPI-linked surface antigens on lipoprotein particles may not be restricted to schistosomes but shared by other blood-dwelling parasites such as *Trypanosoma brucei,* whose VSG surface proteins have been found on host erythrocytes [18]. Conditioned medium of cultivated T. brucei was precleared by high-speed centrifugation and analysed by isopycnic density gradient centrifugation (Figure 8). The major GPI-linked surface antigen VSG221 accumulated in the top fraction of the gradient (culture − PI-PLC, Figure 8A) and was sensitive to treatment with PI-PLC (culture + PI-PLC, Figure 8A). VSG221 was specifically coimmunoprecipitated from the medium by antibodies against mouse ApoA1 (Figure 8B), and was also sensitive to pretreatment with PI-PLC. ApoA1 was chosen because, in contrast to human serum, mouse serum HDL levels are much higher than LDL levels. We concluded, also, that VSG221 associates with lipoprotein particles via its GPI-anchor.

## Discussion

### GPI-Anchored Proteins as Mobile Intercellular Elements

GPI is a complex glycolipid structure that acts as a membrane anchor for many cell surface proteins of eukaryotes [5]. Release of GPI-anchored proteins from the cell surface by specific phospholipases may play a key role in regulation of their surface expression and functional properties. It has been demonstrated that GPI-anchored proteins can undergo intercellular transfer in vitro [13–16]. The present study shows, to our knowledge for the first time, release of endogenous parasite GPI-anchored proteins to human lipoproteins. The lipoprotein complexes formed were then transferred to host cells. In vitro and in vivo data strongly suggest that this process is connected to the pathology of parasite infection. The mechanism of transfer may either be spontaneous diffusion, enhanced by tight binding of human lipoproteins to the schistosome surface [38], or be supported by an unknown mechanism at the plasma membrane or in the endocytic pathway. In *T. brucei,* both GPI-linked surface proteins and LDL share the same endocytic pathway [39], suggesting that transfer takes places in endocytic or recycling organelles. Identification of the mechanism of transfer (which would be a potential drug target) and study of its regulation, are important goals of future research. The principle demonstrated here—GPI-protein transfer between organisms—may also apply to prion diseases such as bovine spongiform encephalopathy and Creutzfeldt-Jakob disease, which are caused by infective GPI-anchored proteins. Intercellular transfer of prion protein has been observed in cell culture [15], but it was claimed that spreading occurs on exosomes [40]. In light of our findings, it seems worthwhile to investigate the role of lipoproteins in prion protein spreading in infected animals.

### Immunologic and Pathophysiologic Aspects

To evade the immune response, parasites have developed various strategies [41], such as the rapid antigenic variation of trypanosomal surface VSG proteins [42]. It is unclear how schistosomes protect themselves from the immune response, in particular adult worms, which can live for years in the portal vein [2]. It appears that release of antigens is a major factor determining the immune response against schistosomes [43,44], but the effects of these released antigens are subject to dispute [45,46]. In general, released antigens have negative modulating effects on the immune system, such as blocking of antibodies and effector cells, induction of tolerance, or activation of suppressor cells. Also, it has been noted that the immune response against a lipopeptide construct that can bind to surface lipids is different from that against the corresponding free peptide for both a viral [47] and a schistosomal antigen [48]. We also speculate that travelling on a lipoprotein surface would change the antigenic properties of a protein, although so far we have found only GPI-linked proteins and other lipidated proteins (Hedgehog and Wingless [20]) on lipoprotein surfaces. Since these proteins are also normally found on cell surface lipids or membrane fragments, uptake, processing, and presentation by antigen-presenting cells should not be grossly altered.

What seems more important is that spreading of antigens via lipoproteins intermixes two pathways—antigen phagocytosis and lipid turnover—that normally are kept separate from each other. On the one hand, cells that are not specialized in antigen phagocytosis will endocytose schistosomal surface antigens via the LDL receptor pathway. As an example, endocytosis of lipoprotein particles from schistosomiasis patient serum changed the morphology of lysosomes in fibroblast cells, which probably reflects problems in the lysosomal degradation of schistosomal glycoconjugates. Similar lysosomal phenotypes are observed in glycolipid storage diseases such as Fabry's and Gaucher's disease, with accumulations in kidney tubule cells and cardiac muscle [49], or in macrophages [50], respectively. In schistosomiasis, lysosomal abnormalities or defects have not been described so far. On the other hand, immune cells that are specialized for endocytosis of antibody-antigen complexes will attack lipoprotein particles. Neutrophils, which are the most important phagocytes of the blood, take up lipoproteins of schistosomiasis patients via FcγRIIa receptors, as demonstrated in this study. This should contribute to the reduction of lipoprotein levels in patients [37], which has the interesting secondary effect of protecting the host from atherosclerosis [51,52]. With the endocytosed lipoproteins, these cells take up large amounts of lipids, giving rise to intracellular accumulation of lipids, shown in Figure 7C and 7D both in vitro and in vivo. Damage induced by the lipid accumulation may contribute to neutrophil apoptosis and low neutrophil counts in schistosomiasis patients [53], supporting immune evasion by the parasite. In support of this, schistosomiasis patient serum promotes neutrophil apoptosis. We can mimic this effect by using normal serum to which anti-ApoB antibodies were added, which effectively is a treatment with a lipoprotein-antibody complex similar to the ones generated in schistosomiasis by antibody-GPI-protein complexes on the lipoprotein particles. Peripheral immunocomplexes in schistosomiasis also act on B cells, which also carry the Fc receptor, to reduce proliferation and MHC class II expression [54]. Other cells that endocytose antigen via Fc receptors are macrophages and dendritic cells, which also are exposed to antigen-loaded lipoproteins. Released schistosomal antigens strongly modulate their behavior to favour a Th-2 type immune response [2]. It is tempting to speculate that, similar to the alterations of lysosomal structure in fibroblast in response to antigen-loaded lipoproteins, antigen processing by these specialized cells might also be altered. Since lysosomal activity is an important parameter influencing efficient antigen presentation [55], this might ultimately contribute to a modified immune response.

## Abbreviations

GPI: glycosyl-phosphatidylinositol
LDL: low-density lipoprotein
mab: monoclonal antibody
PI-PLC: phosphatidylinositol-specific phospholipase C
PMN: polymormorphonuclear neutrophils
GP: schistosomal glycoprotein
